# Supplementary material for: ProteinShader: illustrative rendering of macromolecules
Source: BMC Struct Biol. 2009 Mar 30;9:19. doi: 10.1186/1472-6807-9-19 (PMC2672931; doi:10.1186/1472-6807-9-19)
Supplement: Additional file 1 — ProteinShader program without source code. This compressed file contains the complete ProteinShader program including associated libraries, but no source code. A README.txt file gives an overview of the ProteinShader distribution, and the index.html file in the help subdirectory has directions on getting started with the program as well as a set of tutorials. [file 1472-6807-9-19-S1.zip › ProteinShader-beta-0_9_4-binary/help/api/org/proteinshader/structure/exceptions/package-use.html]

Uses of Package org.proteinshader.structure.exceptions (ProteinShader API)


|  |  |  |
| --- | --- | --- |
| **Overview** \| **Package** \| Class \| **Use** \| **Tree** \| **Deprecated** \| **Index** \| **Help** | |  |
| PREV   NEXT | **FRAMES**    **NO FRAMES**     **All Classes** |  |


---


## **Uses of Package org.proteinshader.structure.exceptions**

| Packages that use org.proteinshader.structure.exceptions | |
| --- | --- |
| **org.proteinshader.gui.components.controlpanel** | Holds the JPanels used in the right-side retractable control panel of the GUI. |
| **org.proteinshader.structure** | Holds the classes that store information from a Protein Data Bank file: Structure, Model, Chain, AminoAcid, Heterogen, Water, Atom, Bond, Helix, BetaStrand, Loop, *etc*. |
| **org.proteinshader.structure.factory** | Holds the factory classes needed for generating the Segment objects that hold the information needed for rendering ribbon and tube segments. |
| **org.proteinshader.structure.visitor.modifiers** | Holds helper classes for the ModifierVisitor class, which is used to modify Drawable objects (Atoms, Bonds, and Segments). |

Classes in org.proteinshader.structure.exceptions used by org.proteinshader.gui.components.controlpanel

****ColorOutOfRangeException****
  
          Used to report that a color value is out of range.

Classes in org.proteinshader.structure.exceptions used by org.proteinshader.structure

****ColorOutOfRangeException****
  
          Used to report that a color value is out of range.

****InvalidIDException****
  
          Used to report that an ID is invalid or missing.

****InvalidRegionException****
  
          Used to report that a Region (Helix or BetaStrand) is invalid because
its start and end Residue IDs could not be matched to an existing
sequence of Residues in a Chain.

****MissingAATypeException****
  
          Used to report that an AminoAcid did not have a type specified.

****MissingAtomTypeException****
  
          Used to report that an Atom did not have a type specified.

****MissingHetNameException****
  
          Used to report that a heterogen name is missing.

Classes in org.proteinshader.structure.exceptions used by org.proteinshader.structure.factory

****SegmentException****
  
          Used to report an error while trying to create a Segment object.

Classes in org.proteinshader.structure.exceptions used by org.proteinshader.structure.visitor.modifiers

****ColorOutOfRangeException****
  
          Used to report that a color value is out of range.

---


|  |  |  |
| --- | --- | --- |
| **Overview** \| **Package** \| Class \| **Use** \| **Tree** \| **Deprecated** \| **Index** \| **Help** | |  |
| PREV   NEXT | **FRAMES**    **NO FRAMES**     **All Classes** |  |


---

# *Copyright © 2007-2008*
